# Single Cell Genomics of Uncultured, Health-Associated *Tannerella BU063* (Oral Taxon 286) and Comparison to the Closely Related Pathogen *Tannerella forsythia*

**DOI:** 10.1371/journal.pone.0089398

**Published:** 2014-02-14

**Authors:** Clifford J. Beall, Alisha G. Campbell, Daniel M. Dayeh, Ann L. Griffen, Mircea Podar, Eugene J. Leys

**Affiliations:** 1 Division of Oral Biology, College of Dentistry, The Ohio State University, Columbus, Ohio, United States of America; 2 Biosciences Division, Oak Ridge National Laboratory, Oak Ridge, Tennessee, United States of America; 3 Genome Science and Technology Program, University of Tennessee, Knoxville, Tennessee, United States of America; 4 Division of Pediatric Dentistry and Community Oral Health, College of Dentistry, The Ohio State University, Columbus, Ohio, United States of America; J. Craig Venter Institute, United States of America

## Abstract

The uncultivated bacterium *Tannerella BU063* (oral taxon 286) is the closest relative to the periodontal pathogen *Tannerella forsythia*, but is not disease-associated itself. Using a single cell genomics approach, we isolated 12 individual BU063 cells by flow cytometry, and we amplified and sequenced their genomes. Comparative analyses of the assembled genomic scaffolds and their gene contents allowed us to study the diversity of this taxon within the oral community of a single human donor that provided the sample. Eight different BU063 genotypes were represented, all about 5% divergent at the nucleotide level. There were 2 pairs of cells and one group of three that were more highly identical, and may represent clonal populations. We did pooled assemblies on the nearly identical genomes to increase the assembled genomic coverage. The presence of a set of 66 “core” housekeeping genes showed that two of the single cell assemblies and the assembly derived from the three putatively identical cells were essentially complete. As expected, the genome of *BU063* is more similar to *Tannerella forsythia* than any other known genome, although there are significant differences, including a 44% difference in gene content, changes in metabolic pathways, loss of synteny, and an 8–9% difference in GC content. Several identified virulence genes of *T. forsythia* are not found in *BU063* including *karilysin*, *prtH*, and *bspA*. The absence of these genes may explain the lack of periodontal pathogenesis by this species and provides a new foundation to further understand the genome evolution and mechanisms of bacterial-host interaction in closely related oral microbes with different pathogenicity potential.

## Introduction

The human oral microbiome contains a significant number of uncultivated organisms that have been identified only by DNA sequences [1], [2]. Such uncultivated organisms may play important roles in health and disease [3].

Chronic periodontitis is a polymicrobial disease that is accompanied by increases in a large number of bacterial species and decreases in others, with uncultured species prominent in both the disease-associated and health-associated groups [3]. The shift in bacterial communities is associated with an increased host inflammatory response that can lead to bone recession and eventual tooth loss.

The genus *Tannerella* of phylum *Bacteroidetes* currently has one named species, *Tannerella forsythia* (previously named *Bacteroides forsythus* and *Tannerella forsythensis*). This oral microbe was shown to be associated with periodontitis along with *Porphyromonas gingivalis* and *Treponema denticola* [4] (*n.b.* a comprehensive analysis reveals many additional periodontitis-associated taxa [3]). *T. forsythia* has been shown to cause periodontitis-like bone loss in both mouse and rat models [5], [6] and a number of putative virulence genes have been identified in its genome [7].

Other uncultured members of the *Tannerella* genus that occur in the oral cavity have been identified by rRNA gene sequences. Of these uncultured species, one designated *Tannerella BU063* (aka Human Oral Taxon 286) is of interest because it was found to be more prevalent in healthy than disease-affected periodontal pockets [8], [9]. *BU063* has been visualized as a segmented rod by microscopy [10]. Additionally, another related species named *Tannerella* Oral Taxon 808 was found to be elevated in periodontitis lesions [3] (our unpublished data). An additional cultured bacterium from the gut has been designated *Tannerella sp. 6_1_58FAA_CT1* and its genome has been sequenced. However it appears to be quite divergent from the oral isolates as demonstrated by the fact that *T. forsythia* and *BU063* have 94% identical 16S rRNAs, while *T. forsythia* and *6_1_58FAA_CT1* have only 86% identity.

The evolution of virulence in human pathogens has been studied extensively by comparative genomics and is often found to involve the acquisition of clusters of genes known as “pathogenicity islands” [11]. Such islands are clusters of genes acquired by horizontal gene transfer that may encode various functions including secretion systems, toxins, iron scavenging, and/or antibiotic resistance [11]. Alternatively, virulence can result from the loss of certain genes, termed antivirulence genes [12].

Single cell genomics is an approach that has been used extensively to study uncultured microbes from a wide range of environments, including the human oral cavity [13], [14], [15]. As part of an effort to obtain a large collection of amplified single cell genomes (SAGs) from oral samples collected from healthy individuals and from periodontitis patients we obtained a set of SAGs that had 16S rRNA gene sequences over 99% identical to *Tannerella BU063*. Their genomes provided the opportunity to gain insights into the genomic determinants of pathogenicity in *Tannerella forsythia* by comparative genomics and help to extend our knowledge of uncultured oral commensal organisms. It is noteworthy in this context that *Tannerella BU063* has been placed on a “most wanted” list of human commensals (OTU_125_V3V5 at http://www.hmpdacc.org/most_wanted/). This list consists of high-priority organisms that have not yet had their genomes sequenced for reference purposes [16].

## Results

### Genome amplification, sequencing, and assembly

We amplified genomic DNA of 327 unselected cells from subgingival plaque of a healthy subject. PCR amplification of the 16S rRNA gene, sequencing, and BLAST search of the CORE oral 16S gene database [2] revealed 12 SAGs that were greater than 99% identical to *Tannerella BU063*. Genomic libraries were prepared from the 12 SAGs using the Nextera library prep kit with multiplex modification. The samples were pooled and sequenced in a single lane of the Illumina HiSeq giving 100 bp paired end reads. The total sequence generated was 5.2×10^10^ bases. We demultiplexed the sequence reads, trimmed them, and assembled the sequences with SPAdes v 2.3 [17]. We then dereplicated and filtered the assemblies as described in materials and methods. Table 1 shows assembly statistics.

**Table 1:** Characteristics of whole genome assemblies derived from individual *Tannerella BU063* cells and combined data from highly identical cells.

Cell #	Contigs (>100 bp)	Total Length, bp	Mean contig size	Max. contig size	N50 Contig	N50 Length	N90 Contig	N90 Length
1	1089	2,409,973	2213	89,442	40	15,243	391	632
2	528	3,145,901	5958	207,881	18	56,975	97	3,824
3	1247	1,617,369	1297	52,561	55	6,290	660	368
4	1290	1,460,671	1132	39,218	76	3,954	755	335
5	779	3,225,702	4140	127,114	21	50,447	145	1,973
6	892	2,553,172	2862	83,095	36	18,857	270	963
7	1287	2,208,786	1716	111,186	53	8,953	572	469
8	1532	1,557,553	1016	23,102	96	3,782	910	320
9	1608	2,948,516	1833	167,492	37	15,762	565	461
10	1527	2,021,332	1323	33,221	88	5,479	812	375
11	1178	2,044,563	1735	86,847	48	9,963	527	464
12	1369	1,739,722	1270	39,570	71	5,915	696	358
1+3 comb.	1337	2,801,227	2095	74,835	45	13,693	491	588
6+7+9 comb.	1573	3,582,672	2277	122,826	42	22,995	442	701
8+11 comb.	1801	2,711,973	1505	75,191	72	8,357	823	414

### Comparison of isolates, completeness estimates, and pooled assemblies

**Figure 1:**
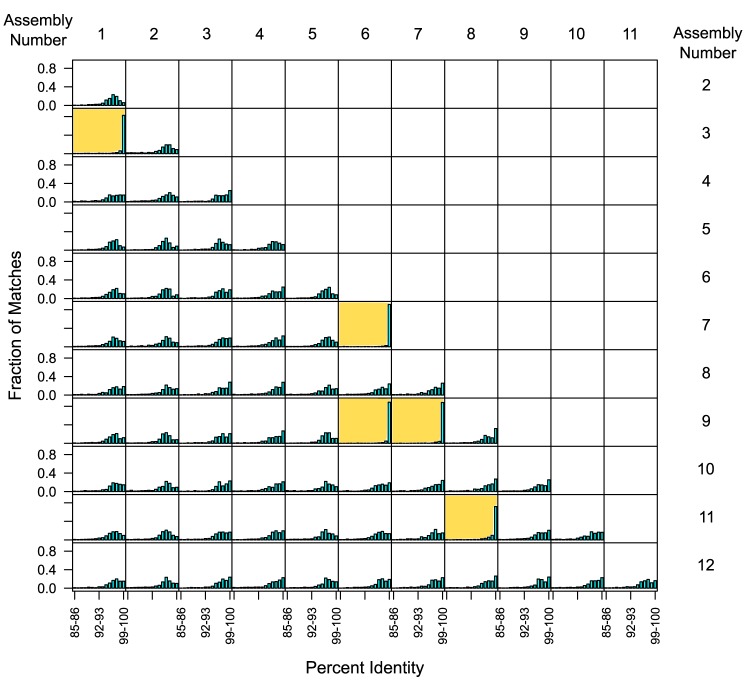
Histograms of blast alignments of all *BU063* single cell assemblies. In each graph, the fraction of the total length aligned is graphed by percent nucleotide identity. Comparisons that showed a high degree of alignments with over 99% identity are highlighted in yellow.

To determine how similar assemblies from individual cells were to each other, we aligned each sequence assembly to the other 11 using BLAST [18], and then determined the best alignments across the entire assembled sequence. Figure 1 shows histograms for all comparisons, calculating the fraction of each genome assembly that aligns at the indicated percentage identities. For 56 of the comparisons, the percentage identity was distributed between 91–100%, however for 5 of them a large fraction of sequence identity was over 99%. These 5 comparisons are highlighted in the figure. They involve one set of 3 cells: cells 6, 7, and 9, and the two pairs: cells 1 & 3 and 8 & 11. It is apparent that a sample from a single individual contains multiple strains of this bacterial species that have substantial nucleotide divergence between their genomes.

It has been observed that multiple displacement amplification can amplify different regions of the genome unevenly and is random in terms of which genomic regions are under or over-amplified [19]. If we assume that these high-identity genome assemblies represent random fractions of a complete genome, we can estimate the full genome size based on overlap. We therefore compared the total assembled lengths to the observed overlap for the five pairs of SGAs that were identical or nearly identical. From this we predicted the genome size as the product of the two total lengths divided by the overlap. Table 2 shows that this predicts a genome of 3.44 to 4.07 Mb, close to the known genome size of *Tannerella forsythia*, 3.4 Mb. We note that the presence of conserved repeated sequences would increase this estimated genome size, making it an upper-limit estimate.

**Table 2:** Prediction of *Tannerella BU063* full genome size based on overlap of partial genomes.

Assembly 1	Assembly 2	Total Length 1	Total Length 2	Overlap	Predicted Genome
Cell 1	Cell 3	2.41 Mb	1.62 Mb	1.13 Mb	3.44 Mb
Cell 6	Cell 7	2.55	2.21	1.61	3.50
Cell 7	Cell 9	2.21	2.95	1.70	3.82
Cell 6	Cell 9	2.55	2.95	1.85	4.07
Cell 8	Cell 11	1.56	2.04	0.86	3.70

In an attempt to derive more complete genomes, we pooled the raw data from the groups of cells that had high identity and re-assembled. The attributes of the assemblies from the combined data are shown in the last three rows of Table 1. They extended the total length of contigs over the assemblies from individual cells. The three combined assemblies and the assemblies from cells no. 2 and no. 5 had total lengths that were similar to the predicted genome size, so they were used for gene annotation by the Integrated Microbial Genomes-Expert Review (IMG-ER) system [20] followed by manual adjustments.

### Presence of conserved core genes

As an independent measure of the completeness of the genomes, we examined the presence of a set of 66 bacterial core housekeeping genes by comparison to the orthologous genes in *T. forsythia* (Supplemental Table S1 in File S1). As shown in Table 3, three of the assemblies had homologs to all 66 core genes, indicating they are likely to be nearly complete genome representations. The other two are less complete. Supplemental Table S2 in File S1 shows the location of the orthologous genes within the *BU063* assemblies.

**Table 3:** Core gene presence in *Tannerella BU063* genome assemblies.

Assembly	Genes Present (of 66)
Cell 2	66
Cell 5	66
1+3 comb.	63
6+7+9 comb.	66
8+11 comb.	60

### Gene content comparisons

**Figure 2:**
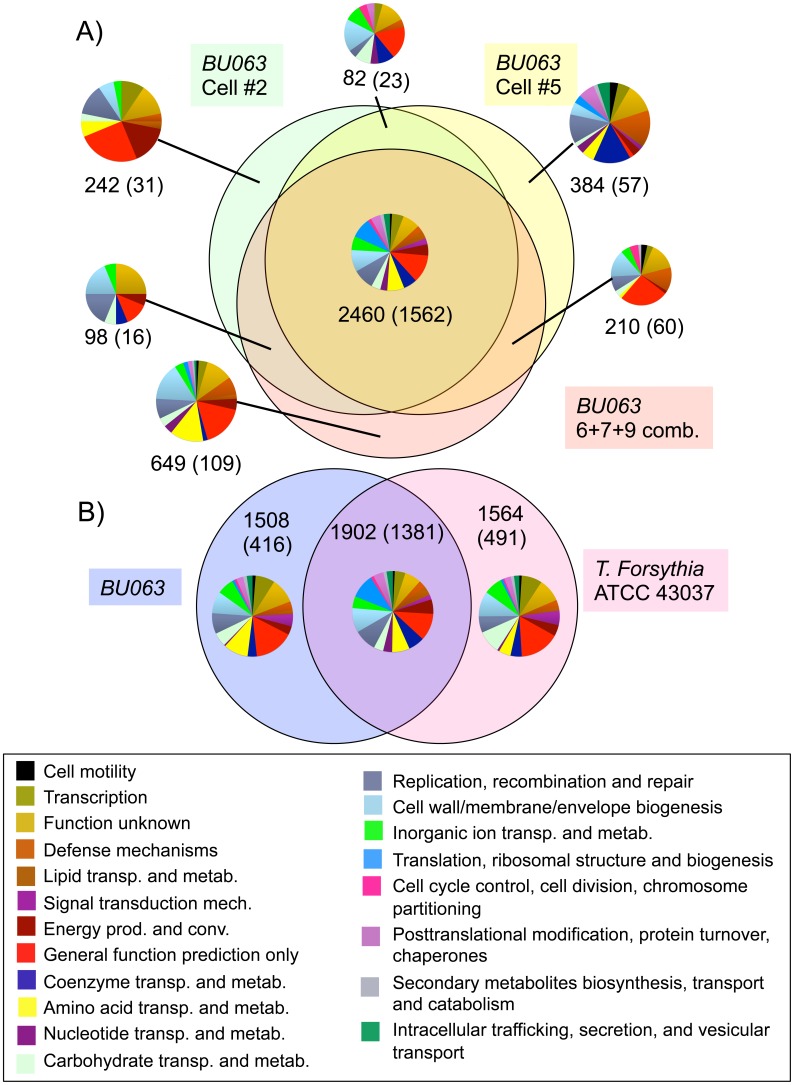
Venn diagrams showing numbers of shared protein-coding genes between various assembled genomes. The first number is the total number of genes in each section, while the number in parentheses is the number of genes that were assigned to COGs. The distribution of COG categories is shown as a pie chart with colors as in the legend **A)** Venn diagram for the three most complete assemblies of *Tannerella BU063*. **B)** Venn diagram showing average numbers comparing *BU063* to *Tannerella forsythia*.

With these nearly complete genome sequences and annotations prepared with the IMG tool, we were able to make inferences about function and comparisons to *Tannerella forsythia*. Figure 2A shows a Venn diagram comparing gene content between the three *BU063* genome assemblies containing all 66 housekeeping genes. A set of 2460 genes are found in all three assemblies, which is on average 78.6% of the total of any one genome. It is notable that genes that are shared between genomes are much more likely to be identifiable as exemplified by membership in a Cluster of Orthologous Groups (COG) [21], for instance 63.5% of the core genes shared by all three genomes are associated with COGs, 25.4% of genes shared by two of the three, and 15.5% of genes that are limited to a single genome. A functional breakdown of gene content by COG categories did not show clear patterns of difference in the three genomes.

Figure 2B shows a comparison of gene content between *Tannerella BU063* and the single sequenced genome of *Tannerella forsythia* (numbers averaged from the three *BU063* genomes). On average 55.8% of genes in *BU063* are shared with *forsythia*. As with the inter-strain comparisons above there was a much greater ability to assign the shared genes to COGs, though there was less variability in the categories between the different gene sets.

### GC content and synteny

**Figure 3:**
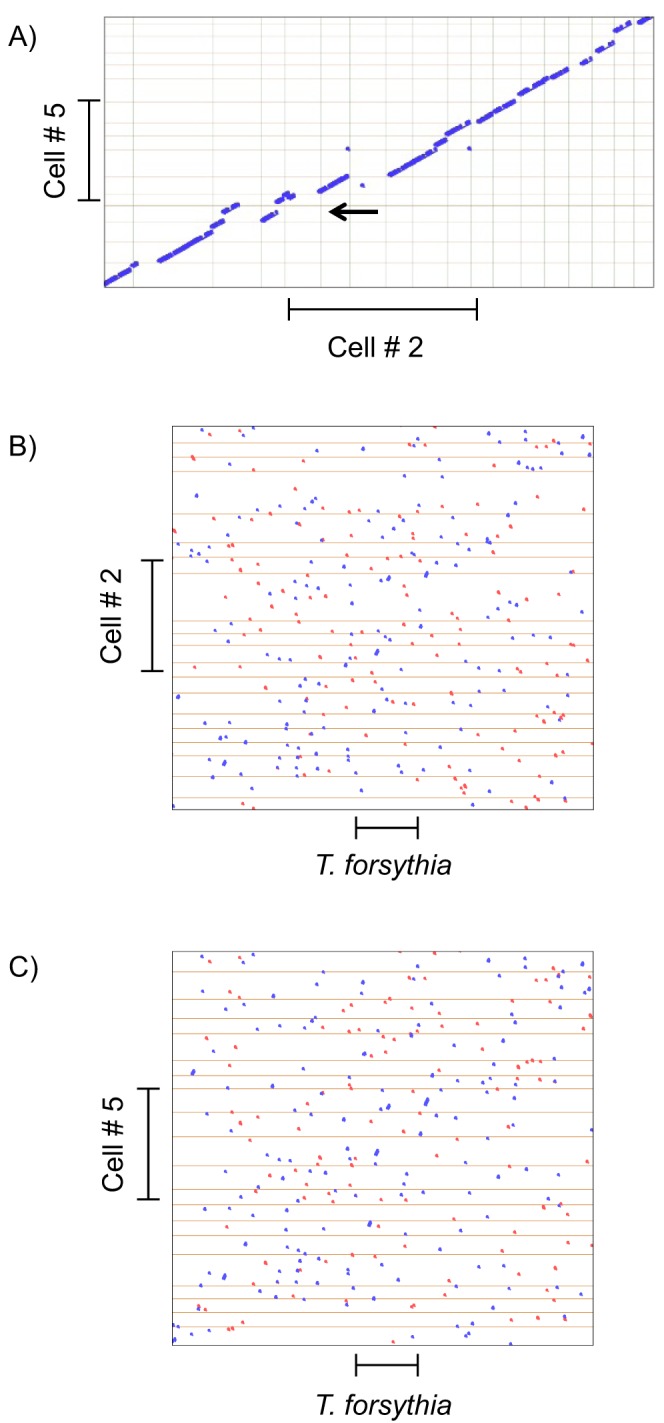
Promer dot plots showing extent of synteny between genomes. The program compares amino acid translations of the genomic sequences. Blue dots indicate identity from + to + or − to − strands, red dots indicate identity from + to − or − to +. The gray grid lines indicate the borders of contigs in the draft assemblies. The bars indicate lengths of 500 kb. **A)** Plot of the 20 largest contigs of *BU063* cell no. 5 assembly against the 20 largest contigs of the cell no. 2 assembly. In either case about half the genome is represented. Note that the program attempts to flip and order contigs to generate a diagonal but this is not fully possible because of missing regions. One non-syntenous region is shown with the arrow. **B)** Plot of the 20 largest contigs of cell no. 2 vs. *T. forsythia* **C)** Plot of the 20 largest contigs of cell no. 5 vs. *T. forsythia*.

Interestingly, although the two species share over half of their genes and occupy the same habitat in the oral cavity there are some major differences in their genomes. A particularly striking statistic is the GC content of the respective genomes. While the five relatively complete *BU063* genomes contain 55 or 56% GC, the *T. forsythia* ATCC 43037 genome is only 47% GC. A further difference is a lack of synteny between *BU063* and *T. forsythia*. As seen in Figure 3A different strains of *BU063* have syntenous regions extending many tens of kilobases. However, Figure 3B and 3C indicate the order of genes between *T. forsythia* and *BU063* has been extensively rearranged. Closer examination shows that short clusters of genes are preserved between *T. forsythia* and *BU063*. These clusters usually are oriented on the same strand and likely represent operons.

### Comparisons of BU063 and T. forsythia for specific functions and potential virulence determinants

We predicted metabolic differences between *Tannerella forsythia* and *Tannerella BU063* using the three most complete genome assemblies of the latter. A listing of differentially present genes is presented in supplemental Table S3 in File S1. In amino acid biosynthesis, *T. forsythia* has a number of genes for arginine biosynthetic enzymes while *BU063* lacks them. It should be noted however that arginine synthetic pathways are complex and not every component was positively identified in *T. forsythia*. *T. forsythia* contains a glutaminase that is absent in the related organism. Conversely, we found that *BU063* encodes genes for biosynthesis of branched chain amino acids, tryptophan, glutamine, and cysteine that are lacking in *T. forsythia*. Interestingly, cysteine synthase enzymes similar to the ones in *BU063* use H_2_S as a substrate, which might be produced by sulfate-reducing bacteria in the periodontal pocket [13]. Additionally, *BU063* has a number of reductase genes that may be involved in respiratory reduction of nitrate, and could provide ammonia that might be utilized by glutamine synthase. *T. forsythia* has two copies of an putative operon encoding genes involved in 1,4-dihydroxy-2-naphthoate biosynthesis, while *BU063* lacks these. 1,4-dihydroxy-2-naphthoate is a key intermediate in production of quinone electron acceptors. Both organisms appear to have enzymes to convert 1,4-dihydroxy-2-napthoate to menaquinone. *T. forsythia* requires the peptidoglycan precursor N-acetyl muramic acid (NAM) for growth [22], and lacks two enzymes required to convert UDP-N-acetyl-D-glucosamine to UDP-N-acetyl-muramic acid. These two enzymes are present in *BU063*, hence we hypothesize that *BU063* may not require exogenous NAM for growth.

Another difference between the species was evident in the genes for rotary ATPases. *T. forsythia* contains genes for both F0F1 and vacuolar/archaeal (V/A) type rotary ATPases, while *BU063* possesses only the V/A type. The locations of these genes are given in supplemental Table S4 in File S1. Although the V/A type ATPase functions mostly as an ion pump in eukaryotes, in bacteria and archaea both types of ATPase are reversible and capable of either exporting ions or generating ATP in response to ion gradients [23]. To determine the frequency of different ATPase genotypes in bacteria we performed a survey of all the genomes in IMG (accessed April 1, 2013) with the phylogenetic occurrence profiler for COG groups corresponding to the subunits of either ATP synthase. The results indicated that genomes with only the V/A-type ATPase are rare. Out of the sequenced genomes analyzed, 4227 had only the F0F1 type, 614 had both, 255 had only the V/A type, and 33 had none. Organisms with only the V/A-type were concentrated in a few taxa, including the genera *Porphyromonas*, *Alistipes*, *Chlamydia*, *Anaerococcus*, *Parvimonas*, *Peptoniphilus*, *Borrellia*, *Spirochaeta*, and *Treponema*, and the phyla *Deinococcus-Thermus* and *Synergistetes*. It is notable that many of these genera are found in the oral cavity.

A number of genes have been linked to pathogenicity in *T. forsythia* [7]. Therefore it is of interest to examine whether these genes have orthologs in *Tannerella BU063*. We searched for homologs to some of the most studied genes in *T. forsythia* using blastp against annotated genes in *BU063*, following up with tblastn against the DNA sequences if blastp gave no result. Detailed information on these putative virulence genes from *T. forsythia* is given in supplemental Table S5 in File S1, and details on the homologs from *BU063* are in supplemental Table S6 in File S1.

PrtH/FDF is a cysteine protease of *T. forsythia* that has been shown to cause detachment of adherent cultured cells and expression of the pro-inflammatory cytokine IL-8 [24], [25], [26]. PrtH is a novel type of protease without detectable identity to other known enzymes, though its folding may be somewhat similar to the gingipains of *P. gingivalis* [27]. We did not find homologs of *prtH* in *BU063*.

Karilysin is a metalloprotease of *T. forsythia* that has been implicated as a virulence factor due to its ability to cleave and inactivate several components of complement [28]. The karilysin protein contains an enzymatically active domain and a C-terminal Por secretion system sorting domain [29]. The catalytic domain is similar to a small number of bacterial enzymes, and also quite similar to matrix metalloproteases of eukaryotic cells. Although the C-terminal Por secretion domain matches a number of proteins in *BU063*, the catalytic domain does not. We therefore conclude that *BU063* does not encode a functional karilysin.

BspA is a cell surface protein of *T. forsythia* that contains a number of domains including 14 23-amino acid leucine rich repeats [30] followed by a *Bacteroidetes*-associated carbohydrate-binding often N-terminal (BACON) domain [31], a bacterial immunoglobulin-like domain (group 2), and (as with karilysin) a C-terminal Por secretion system domain. BspA has been linked to pathogenicity of *T. forsythia* through several lines of evidence. It is needed to produce alveolar bone loss in mice [5], it is required for invasion of epithelial cells by the bacterium [32], and it is needed for induction of IL-8 production mediated through Toll-like receptor 2 [33]. We performed searches of the assembled genomes with the BspA protein. Although there were matches to all of the domains separately, there were no cases in which all 4 domains were present in a single protein/gene in any of the *BU063* genome assemblies.

*T. forsythia* contains a sialidase gene, *nanH*, that is part of a nine gene complex including a putative sialic acid transport system [34]. The NanH protein has been implicated in attachment to epithelial cells and glycoprotein-coated surfaces [35], [36]. Searches found no evidence for the NanH sialidase in *BU063*. While there were some distant matches in *BU063* (<30% identity) to the TonB dependent receptor and SusD family components of the transport system, those similarities were much less than the same genes showed to *Bacteroides* species (69% identity).

Certain other genes that have been implicated in the virulence of *T. forsythia* do have orthologs in *BU063*. *T. forsythia* possesses an S-layer outside its outer membrane, that is composed primarily of two glycoproteins, TfsA and TfsB [37]. The two proteins show some similarity to each other and to proteins from other *Bacteroidetes* such as *Parabacteroides disatonis* [38]. The structure of the glycosyl moiety that modifies these proteins has been determined and the *wecC* gene, a putative nucleotide-sugar dehydrogenase, has been shown to be involved in the glycosylation [39]. The *wecC* gene is part of a cluster of genes with predicted glycosylation functions [39]. Mutants in the S-layer proteins have been shown to affect binding to epithelial cells, coaggregation with other oral bacteria, and serum resistance [40], [41], while mutants in *wecC* increase biofilm formation [42]. WecC also participates in synthesis of an O-linked glycan that suppresses T-helper 17 cell activation [43]. The *tfs* genes each have orthologs in the *BU063* assemblies that are 54-60% identical (as opposed to the *Parabacteroides* proteins that are less than 30%) and have the same tandem arrangement. We found sequences with high identity to *wecC* in all the *BU063* assemblies by TBLASTN search, but in 3 of the 5 the identical regions were short and fragmented into multiple contigs. The combined 6/7/10 assembly had the most intact assembly for this genomic region. The *wecC* gene is highly conserved with over 94% amino acid identity between *T. forsythia* and *BU063*, and the subsequent gene in the cluster, a predicted UDP-N-acetylglucosamine 2-epimerase has 92% identity between the two species. Three glycosyl transferase genes found in *T. forsythia* have counterparts in *BU063* that are 49%, 52% and 71% identical to various genes found in the *BU063* genomes, while an acetyl transferase gene that is clustered with the others in *T. forsythia* does not have a close match in *BU063*.

It has been suggested that production of the toxic compound methyl glyoxal by *T. forsythia* might lead to disease pathogenesis [44]. We found that *BU063* has a gene that is 90% identical to the *mgsA* methyl glyoxal synthase of *T. forsythia*.

### Bioinformatic identification of possible virulence determinants

To identify previously unknown genes that might be involved in pathogenesis in *T. forsythia*, we pursued a broader approach. As mentioned earlier, we had found a large number of genes that were present in *T. forsythia* and not *BU063*. To focus on genes that might be functional in periodontitis, we used previous data we had derived on association of various species with periodontitis [3], [45] and the substantial number of bacterial genomes that have been sequenced as part of the Human Microbiome Project [46]. We required that genes be present in *T. forsythia* as well as in genomes of other sequenced *Bacteroidetes* that are significantly associated with periodontitis but absent in *BU063*. The species used were: *Porphyromonas gingivalis* (three genomes), *Porphyromonas endodontalis* (one), *Prevotella intermedia* (three), *Prevotella denticola* (two), *Alloprevotella* (formerly *Prevotella* [47]) *tannerae* (one), and *Bacteroidetes sp. F0058* (aka human oral taxon 274, one genome). By the criteria used (see Materials and Methods) 236 genes from *T. forsythia* were identified (supplemental Table S7 in File S1), and of these 163 were assigned to COGs. A number of the COG assignments suggested possible functions in pathogenesis, for instance 19 genes in COG category K: transcription, 11 genes in category M: cell wall and envelope biogenesis, seven in category T: signal transduction, and one in category V: defense mechanisms. These are listed in supplemental Table S8 in File S1. Other genes with possible interest were two adjacent genes (loci BFO_0532/0533) that were similar to the N-terminus and C-terminus of the HipA gene product that is involved in regulation of persister cell formation in *E. coli* [48].

**Figure 4:**
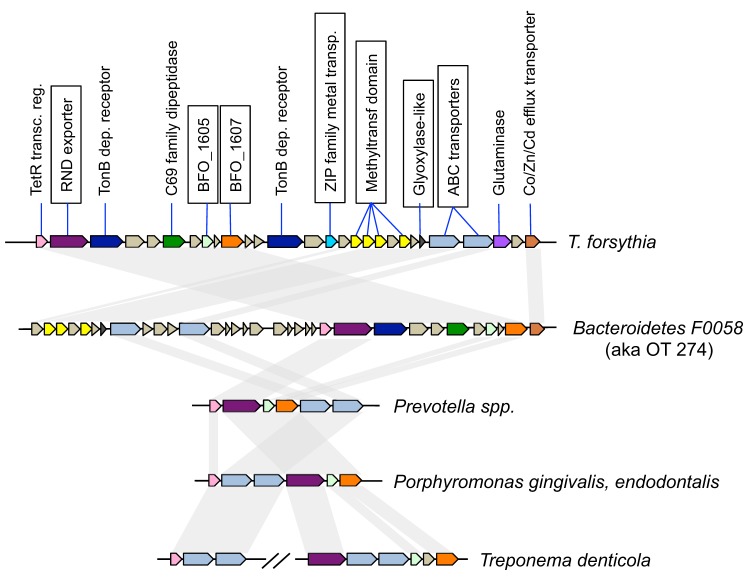
Genome maps of a gene cluster in *T. forsythia* and other organisms. The cluster was identified by a bioinformatics screen for possible virulence genes, identifying genes with homologs in *T. forsythia* and at least one of six *Bacteroidetes* organisms associated with chronic periodontitis, but absent from the five longest *BU063* assemblies (protein BLAST results with cut-offs 30% identity and e-value 10^−5^). A cluster of genes including many with apparent cell-surface function was identified within the results. The predicted gene functions are shown above the *T. forsythia* map, with colors indicating conserved genes in the other organisms. The boxed genes are more similar to the other periodontitis-associated organisms than to *BU063*. Gray bars in the background show the pattern of sequence conservation by connecting genes to homologous genes in the other organisms.

Because virulence genes are often clustered in pathogenicity islands, we examined the distribution of the putative virulence-associated genes and identified 6 clusters (Table 4). Three of the clusters were for functions that have been previously discussed here, for subunits of F0F1 ATPase and two duplicated clusters of menaquinone biosynthetic enzymes. Three additional possibly relevant clusters of genes were found. Of potential interest is a set of genes that span from locus BFO_1597 to BFO_1620, containing a number of membrane and transport proteins, including two ATP-binding cassette (ABC) transporters and a Resistance-Nodulation-Cell Division (RND) family exporter (Figure 4, supplemental Table S9 in File S1). Although the ABC transporters were not identified in the initial screen because of weak similarity to genes in *BU063*, close investigation showed they were much more similar to genes in periodontal pathogens (<35% amino acid identity vs. >60%). A large number of genes in the cluster are highly similar to a cluster of genes in the periodontitis-associated bacterium *Bacteroidetes F0058* (oral taxon 274). Interestingly, six of the genes including the RND exporter, the ABC transporters, a transcription factor and two genes of unknown function were also clustered in all of the periodontitis-associated organisms except possibly *Alloprevotella tannerae* where the draft genome assembly is fragmented in that area. In fact genes with more similarity to *T. forsythia* than *BU063* were even found in the periodontitis pathogen *Treponema denticola*, a spirochete, though the cluster is broken into two parts in this bacterium. The arrangements of the clustered genes in various genomes are depicted in Figure 4.

**Table 4:** Clusters of genes present in *T. forsythia* and other pathogens but missing from *BU063*.

New locus tags	Former locus tags	Number identified/total	Putative function
BFO_0656- 0664	TF1669-1677	8/9	F1F0 ATP synthase
BFO_1596-1620	TF2603-2629	11/25	Hypothetical cell surface-associated
BFO_1951-1955 and 3197-3201	TF2947-2951 and 0864-868	5/5	Quinone cofactor synthesis (repeated)
BFO_2956-2965	TF0625-0634	6/10	Hypothetical cell surface-associated
BFO_3251-3271	TF0915-0934	12/19	Unknown function

## Discussion

We described here the core genome of an uncultivated bacterial species related to a well-known oral pathogen from the human oral microbiota. The presence of the 66 core genes in three genome assemblies suggests that we have a nearly complete picture of this organism's core genome. This completeness is in contrast to some earlier single cell genome studies and is likely due to several factors including increased depth of sequencing, the SPAdes assembly program geared towards single cell data, and the availability of multiple amplified genomes of the same species that complemented each other.

A major impetus to sequencing these genomes was to compare a less pathogenic relative (*BU063*) to the periodontal pathogen, *Tannerella forsythia*. In previous cases, comparative genomics of pathogens and non-pathogenic relatives have identified specific genes that contribute to virulence, and often these genes are part of clusters designated as “pathogenicity islands” [11]. However, in some cases the loss of “antivirulence” genes can also contribute to the evolution of virulence [12]. In the case of *BU063* and *T. forsythia*, although the two species are related they are divergent in both gene content (Figure 2) and gene order (Figure 3). Therefore it is not possible to say from this study if either the pathogenicity island or antivirulence gene loss mechanism was active in acquisition of virulence by *T. forsythia*. It might be possible to gain further insight by studying multiple isolates of *T. forsythia* to correlate possible variations in virulence with genomic variation. Until now, only one *T. forsythia* genome sequence has been made public.

A surprising outcome of this study was the high level of strain polymorphism in the one species isolated from a single subject. Nucleotide comparisons with BLAST indicated that out of 12 *BU063* isolated cells from a single human subject, there were 8 divergent genotypes (Figure 1). From the high degree of nucleotide divergence, *BU063* was likely acquired by this subject multiple times. The high number of strains is perhaps unexpected given that *BU063* is not normally present at high levels in 16S surveys (0.05% of subgingival bacteria on average [3]). It may indicate that the true diversity of the oral metagenome is substantially higher than indicated by species level OTU analyses. The genomes of the different strains of *BU063* appear to be quite similar in gene content and gene order, as much as can be discerned with draft assemblies (Figure 3).

Comparisons between the genomes of *T. forsythia* and *BU063* revealed many differences, albeit they are more closely related to each other than to other sequenced genomes. Among the differences are wholesale shuffling of gene order, a difference in GC content, and differences in specific genes. Despite the striking difference in GC content, similar variation is seen in other genera within the *Bacteroidetes* phylum. A review of the IMG database showed that GC content for *Bacteroides* species vary from 29% to 48%, *Prevotella* species from 36% to 56%, and *Porphyromonas* species from 44% to 56%. Although GC content is influenced by natural selection in bacteria, the underlying drivers are not completely understood [49]. Among genes absent in *BU063* are several that have been implicated in the virulence of *T. forsythia* such as the genes for the cell surface protein BspA and the proteases PrtH and karilysin. Additionally, though *BU063* has S-layer glycoproteins similar to *T. forsythia*, there are suggestions that there may be differences in their glycosylation, which could be significant in their function [43]. Though these differences may explain the lack of pathogenicity of *BU063*, we have sought additional candidate virulence genes by bioinformatic techniques. Our search was based on the hypothesis that there might be common genes of *Bacteroidetes* that are associated with periodontitis. This search has identified candidate genes that can be studied further, including one gene cluster that appears to be found in a large number of genomes from periodontitis-associated organisms (Figure 4).

There have been a number of attempts to culture *BU063*, and a recent publication described some success in co-culturing it with *Prevotella oris* [50]. One possible explanation for this is the absence of the genes for dihydroxynaphthoate biosynthesis leading to quinone cofactors. The five genes discussed earlier are present in three available genome sequences of *P. oris*. On the other hand, menadione is commonly used as a supplement to bacterial culture media, so it is not obvious that the lack of the pathway should have been a block to previous culture attempts. Conversely, the genome sequences presented here suggest that supplementation of medium with N-acetyl muramic acid should not be required to culture *BU063*, unlike the situation with *T. forsythia*.

In conclusion, we have sequenced a previously uncharacterized member of the oral microbiome, providing a useful reference. Comparisons with the related pathogenic bacterium *T. forsythia* are not definitive because of the relatively high degree of divergence, but give possible hints about gene clusters that may be worth further investigation. Finally we identify a high degree of strain diversity of this one species in the mouth of a single individual, suggesting that the oral microbiome is a more complex ecosystem than revealed by species-level analysis.

## Materials and Methods

### Ethics Statement

This study was specifically approved by the Oak Ridge Sitewide Institutional Review Board (OSIRB) and written informed consent was provided by the donor.

### Sampling, cell isolation, and whole genome amplification

Subgingival plaque was collected from a healthy subject and individual bacterial cells were isolated by flow cytometry. A collection of single cell amplified genomes was created by multiple displacement amplification with Phi 29 DNA polymerase and *BU063* genomes were identified by PCR of a segment of the 16S rRNA gene and direct Sanger sequencing. Detailed methods are given in previous publications [13], [14], which analyzed other members of the collection.

### Sequencing

Sequencing libraries were prepared from whole genome amplifications with the Nextera 1^st^ generation library prep kit according to the manufacturer's protocol (Epicentre, Madison WI). Multiplexing barcodes were added with the Illumina-compatible bar code kit from the same manufacturer during the library amplification step. The libraries were purified with Ampure XP (Beckman-Coulter, Indianapolis) (0.7X) and quantitated by Quant-iT kit (Life Technologies, Grand Island NY). Twelve libraries were pooled and run on one lane of the Illumina HiSeq 2000 (Illumina, La Jolla, CA) with 100 bp paired end reads.

### Sequence assembly and filtering

Demultiplexing of sequence reads was carried out with the Illumina software. Quality filtering and adapter trimming was done with the Trimmomatic 0.20 program (http://www.usadellab.org/cms/index.php?page=trimmomatic) [51] with the parameters ILLUMINACLIP:tmp.fa:2:4:15, LEADING:3, TRAILING:3, SLIDINGWINDOW:4:15, and MINLEN:36.

We experimented with a number of assembly programs, including SOAPdenovo [52], velvet [53], velvet-sc [54], velvet following khmer digital normalization [55], and SPAdes versions 2.2 and 2.3 [17]. SPAdes version 2.3 gave the best results in terms of both N50 and total length assembled, so we used it for further work. We used default parameters with kmer lengths of 31, 59, and 83 nt. For combined assemblies, we simply pooled the trimmed data from the individual cells and assembled in the same manner.

We carried out a number of filtering operations on the assembled contigs. A common observation with single cell amplified genome data has been the presence of contamination from other organisms. We carried out a number of exploratory analyses to search for contamination. We therefore screened the assembled contigs against a number of potential sources (identified *ad hoc*) by nucleotide BLAST searches [18] and removed contigs that matched (over half the contig and e-value <0.01) any of: the human genome, the *E. coli* genome, the *Saccharomyces cerevisiae* nuclear or mitochondrial genome, the PhiX174 genome, or the UniVec database of synthetic vector sequences. These removed between 0.7%–12% (mean 3.1%) of the contigs. We also found that apparently due to a bug in SPAdes 2.3, rare contigs were either exactly duplicated, were substrings of other contigs, or were exact inverted repeats. We found such cases by BLAT [56] searches of the assemblies against themselves and removed them (or half in the case of the inverted repeats). This process removed between 0.9% to 4.9% of the assembled lengths, mean 2.1%. We did additional steps after an initial annotation of the genome. One was to discard contigs that did not contain predicted protein-coding regions. We further used the predicted proteins to do a blastp search against the NCBI nr protein database. 47% of identified genes had a best match with *T. forsythia*. For each assembly the next most frequent organism was 1.3–1.6% of matches. For all assemblies the next 100 most matched organisms were also *Bacteroidetes* species, except for four contigs from the cell #2 assembly containing 7 coding sequences that were highly homologous to a *Chthoniobacter flavus* genome, and a single contig with nine genes from the 6/7/9 combined assembly that was highly similar to *Enhydrobacter aerosaccus*. Following a protocol on the IMG web site (https://img.jgi.doe.gov/er/doc/SingleCellDataDecontamination.pdf), we examined ribosomal RNA encoding sequences. All of these were as expected. We also used a tool on the IMG-ER site that performs a principal coordinates analysis of kmer frequencies to identify 2 contigs that may be derived from plants. We further used the phylogenetic profiler on the IMG site to identify 7 other suspect contigs. All identified contaminant contigs were removed from the final assemblies.

### Genome Annotation and Comparisons

The assembled genomes were submitted to the IMG ER system [20] for gene prediction and annotation. Erroneous gene predictions were corrected manually for some ribosomal protein genes and the S-layer protein genes. These were identified manually by blastx searches and the Artemis program [57]. Genome comparisons were done using various functions of the IMG site.

### Core Gene Analysis

The Human Microbiome Project, through their Data Analysis and Coordination Center (http://www.hmpdacc.org/), have provided a list of 66 core genes that should be present in all bacteria and can be used to check the completeness of genome assemblies. We used this list to extract the set of orthologs from *T. forsythia*, since those should be evolutionarily closest to *BU063* (Supplemental Table S1 in File S1). We used the “compare by sequence” tool on the RAST SEED viewer web application to generate comparisons of *T. forsythia* to each of the 5 annotated assemblies at the protein sequence level.

### Bioinformatic search for putative virulence-related genes

The complete set of 3153 annotated protein-coding genes from *Tannerella forsythia* were used with the gene profile tool of IMG-ER to compare with the five assembled *BU063* genomes and the 11 indicated *Bacteroidetes* periodontitis-associated genomes with cut-off settings of 30% identity and e-value <10^-5^. The results indicating numbers of hits per genome were exported to a spreadsheet. A scoring system was used where hits to species with single available genomes were given one point for presence of the gene, and species with multiple genomes were given fractions of points per genome based on the number. Candidates were genes with at least one point in hits to the periodontitis-related species and without hits in any *BU063* genome.

### Data sharing information

The five long assembled genomes are available on IMG as genome IDs 2523231011, 2523231020, 2523231021, 2523231022, and 2523231032. They are also available in Genbank WGS under BioProject numbers 213303, 222524, 222525, 222526, and 222536. The raw data is available in the NCBI SRA under accessions SRP033391, SRP033413, and SRP033408.

## Supporting Information

File S1 **Excel file containing supplementary Tables S1 through S9.**
(XLSX)
